# Prominent Receptors of Liver Sinusoidal Endothelial Cells in Liver Homeostasis and Disease

**DOI:** 10.3389/fphys.2020.00873

**Published:** 2020-07-21

**Authors:** Ekta Pandey, Aiah S. Nour, Edward N. Harris

**Affiliations:** Department of Biochemistry, Universityof Nebraska, Lincoln, NE, United States

**Keywords:** liver, sinusoidal endothelial cells, scavenger receptors, cell surface receptor, endocytosis, ligand binding

## Abstract

Liver sinusoidal endothelial cells (LSECs) are the most abundant non-parenchymal cells lining the sinusoidal capillaries of the hepatic system. LSECs are characterized with numerous fenestrae and lack basement membrane as well as a diaphragm. These unique morphological characteristics of LSECs makes them the most permeable endothelial cells of the mammalian vasculature and aid in regulating flow of macromolecules and small lipid-based structures between sinusoidal blood and parenchymal cells. LSECs have a very high endocytic capacity aided by scavenger receptors (SR), such as SR-A, SR-B (SR-B1 and CD-36), SR-E (Lox-1 and mannose receptors), and SR-H (Stabilins). Other high-affinity receptors for mediating endocytosis include the FcγRIIb, which assist in the antibody-mediated removal of immune complexes. Complemented with intense lysosomal activity, LSECs play a vital role in the uptake and degradation of many blood borne waste macromolecules and small (<280 nm) colloids. Currently, seven Toll-like receptors have been investigated in LSECs, which are involved in the recognition and clearance of pathogen-associated molecular pattern (PAMPs) as well as damage associated molecular pattern (DAMP). Along with other SRs, LSECs play an essential role in maintaining lipid homeostasis with the low-density lipoprotein receptor-related protein-1 (LRP-1), in juxtaposition with hepatocytes. LSECs co-express two surface lectins called L-Specific Intercellular adhesion molecule-3 Grabbing Non-integrin Receptor (L-SIGN) and liver sinusoidal endothelial cell lectin (LSECtin). LSECs also express several adhesion molecules which are involved in the recruitment of leukocytes at the site of inflammation. Here, we review these cell surface receptors as well as other components expressed by LSECs and their functions in the maintenance of liver homeostasis. We further discuss receptor expression and activity and dysregulation associated with the initiation and progression of many liver diseases, such as hepatocellular carcinoma, liver fibrosis, and cirrhosis, alcoholic and non-alcoholic fatty liver diseases and pseudocapillarization with aging.

## Introduction

The liver is considered a crucial organ of the body due to its involvement in numerous processes, such as metabolism, immunity, detoxification, nutrient storage, among others. The liver is composed primarily of four distinct cell types, differentiated into two categories as parenchymal cells (PC, 60–80%) and non-parenchymal cells (NPC 20–40%). The NPC population is composed of liver sinusoidal endothelial cells/LSECs (50%), Kupffer cells/KCs (20%) and stellate cells (<1%). The remaining NPCs are composed of lymphocytes (25%) and biliary cells (5%) (Racanelli and Rehermann, 2006). The role of hepatocytes, KCs and stellate cells in maintaining liver homeostasis is well documented. However, the LSEC, is the most understudied due to technical challenges in purification and culturing *ex vivo*.

Prof. Eddie Wisse first proposed the ultrastructure of liver sinusoidal endothelial cells (LSEC) in 1970, which differentiated LSECs from KCs and paved the way for future study on LSECs and elucidation of their function (Wisse, 1970, 1972). A few years later, several research groups developed LSEC isolation techniques and identified their role in the uptake of various substances *in vitro* (Seglen, 1976; Smedsrod et al., 1984). In the early 1980s, LSECs were identified as a significant clearance site for blood-borne hyaluronan, which established their role as scavenger cells (Fraser et al., 1981). This led to an increase in interest among scientists from other discipline, such as immunology, virology, cancer, and more to further comprehend the various roles performed by LSECs. This review focuses on the detailed description of LSEC morphology and their scavenger, adhesion and other prominent receptors that define their functional roles in the hematological and hepatic systems during health and disease.

## LSEC Morphology

**FIGURE 1:**
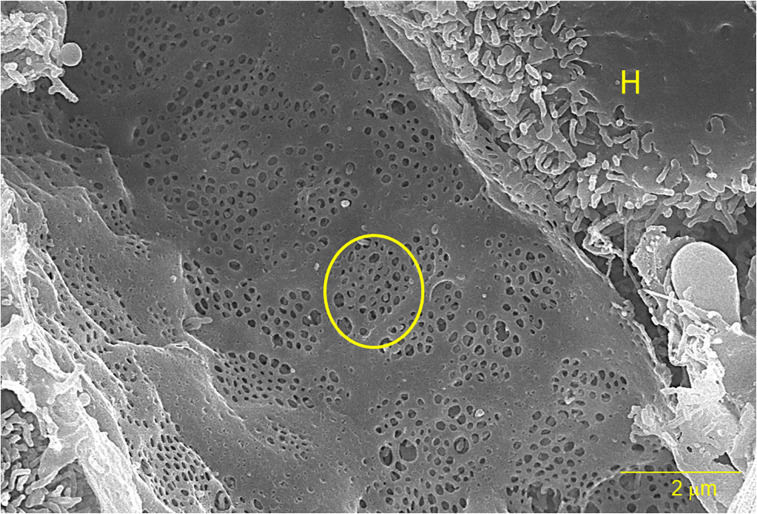
A scanning electron micrograph of a sliced rat liver at 10,000× magnification. The yellow circle outlines one of several sieve plates and the “H” indicates an adjacent hepatocyte. The bar represents 2 μm.

Liver sinusoidal endothelial cells (LSECs) form the inner lining of liver sinusoidal blood vessels or capillary bed which serves as the site for mixing nutrient-rich blood from the hepatic portal vein and oxygen-rich blood from the hepatic artery (Sorensen et al., 2015). Here, the LSECs assist in clearing macromolecular waste (extracellular matrix material and foreign molecules) from the blood and regulate hepatic vascularity. Individual LSEC’s are flat and very small in size, no thicker than 5 μm at the center and 0.3 μm at the periphery (Wisse, 1970, 1972; Smedsrod et al., 1988b; Falkowska-Hansen et al., 2007). Cytoplasmic projections, such as filopodia, lamellipodia, and microvilli are absent in LSECs, giving them a smooth appearance. LSECs contain numerous fenestrae (small open pores) that facilitate the selective exchange of molecules between the blood and underlying stellate and hepatocytes (Wisse et al., 1985; Braet and Wisse, 2002). Fenestrae are located in the cytoplasm of LSECs and usually are 50–200 nm in diameter and organized in groups known as sieve plates (Wisse et al., 1985). They are also distributed individually on the surface of endothelium or organized in a labyrinth or mesh-like structure (Taira, 1994; Braet et al., 2007, 2009; Figure 1). LSECs do not have a basement membrane or basal lamina and diaphragm, permitting direct access of solutes to the perisinusoidal space or the Space of Disse. The lack of a basal lamina has also been identified in several animals, such as chickens and bony fish (Fraser et al., 1986; Eng and Youson, 1992). The Space of Disse hosts the stellate cells as well as hepatocyte microvilli. During fibrosis or chronic inflammation, activated stellate cells contribute to the deposition of extracellular matrix in the Space of Disse, forming a continuous basal membrane. This new basal lamina is mostly composed of collagen that inhibits the permeability of the Space of Disse and reduces the solute exchange between the parenchyma and blood (Han et al., 2001; Mak et al., 2012).

LSEC’s are unique because of their plentiful fenestrae. The LSEC fenestrae can change their diameter in response to the cellular environment (Wisse et al., 1996; Braet and Wisse, 2002). The mechanism for fenestral contraction and dilation was first discovered by researchers in the 1980s and is now known as a dynamic process (Monkemoller et al., 2015; Zapotoczny et al., 2019). Fenestrae is surrounded by actin filaments, suggesting the role of cytoskeleton is central in the formation and maintenance of fenestrae. This idea was further supported by several researchers in the successive years as immunofluorescence microscopic study substantiated the presence of actin, myosin, microtubule and calmodulin as necessary in forming these structures (Wisse, 1970; Braet and Wisse, 2002). Actin, myosin, and calmodulin influence the diameter of each pore and determine how long it persists. Serotonin has also been found to regulate contraction of fenestrae by increasing the intracellular calcium concentration (Gatmaitan et al., 1996; Yokomori, 2008; Furrer et al., 2011). Increase in the thickness of LSECs, unexpected formation of basal lamina and reduction in the number of pores is known as defenestration. Defenestration of LSECs gives rise to pseudocapillarization, a disorder evident in liver fibrosis, atherosclerosis and other aging associated diseases (Le Couteur et al., 2001, 2007). Fenestrae is maintained cooperatively by hepatocytes and stellate cells mediated paracrine and autocrine signaling (DeLeve et al., 2004). Vascular endothelial growth factor (VGEF), a hormone that functions in blood vessel growth, promotes paracrine signaling from hepatocytes and stellate cells stimulates autocrine signaling of nitric oxide (NO) from LSECs. Animals lacking VEGF tend to exhibit increased defenestration in their LSECs, suggesting that this molecule plays a mechanistic role in either their formation or maintenance (Kus et al., 2019).

Defenestrated LSECs results in the reduction of hepatic uptake of lipoproteins and is one of the causes of hyperlipoproteinemia (Fraser et al., 2012). Similarly, defenestration has also been observed to play an important role in the progression of non-alcoholic fatty liver disease (NAFLD). Miyao et al. demonstrated that since LSECs are a “gatekeeper” for the liver parenchyma, their injury during early stages of NAFLD determines the severity of subsequent injury and progression of NAFLD (Miyao et al., 2015; Shetty et al., 2018). In the past few years, some of this data has been called into question in that some have observed no difference in NAFLD and fenestration levels of LSECs and that LSECs have a more calming effect on injured hepatocytes and activated stellate cells (Kus et al., 2019). This idea is reinforced in a study of autoimmune hepatitis in which human LSECs in biopsies from young people less than 20 years old were assessed for damage and the formation of a basement membrane. Defenestration did occur in about half of the subjects, but little to no basement membrane formed in the Space of Disse suggesting that LSECs have regenerative properties and may soften the assault on the liver (Lotowska et al., 2018). While there was no causative relationship proven, the jury is still out on whether progressive NAFLD is associated with defenestration.

Another salient characteristic of LSECs that differentiate them from other endothelial cells is their higher endocytic ability. LSECs only make up about 3% of total liver volume, however, they contribute to about 45% of pinocytic vesicles in the liver (Blouin et al., 1977). A study was conducted to elucidate the mechanism of cross presentation by LSECs as a link was found between endocytosis and antigen presentation (Burgdorf et al., 2008). This study reported that LSECs were most efficient at internalizing circulating antigen in the blood as compared to dendritic cells (DC) or macrophages of the spleen as well as by KC and DC of the liver (Schurich et al., 2009). LSECs are well equipped with high affinity endocytic scavenger receptors and lysosomal activity that helps in the internalization and catabolization of a large number of waste substances (Table 1) as well as small colloidal particles (Knook and Sleyster, 1980; Juvet et al., 1997; Kawai et al., 1998; Elvevold et al., 2008a).

**TABLE 1:** Receptors expressed by LSECs and their known ligands.

Receptor	Ligands	References
SR-A1/SR-A1.1	Ac-ox LDL	Suzuki et al., 1997; Kunjathoor et al., 2002
	β-amyloid fibrils	El Khoury et al., 1996
	Advanced glycation end products	Araki et al., 1995
	Lipopolysaccharide	Hampton et al., 1991
	Lipoteichoic acid	Dunne et al., 1994
	Malondialdehyde-acetaldehyde-serum albumin	Duryee et al., 2004
SR-B1/SCARB1	Unmodified LDL	Kozarsky et al., 1997
	Oxidized LDL	Kozarsky et al., 1997
	VLDL	Kozarsky et al., 1997
	HDL	Acton et al., 1996; Kozarsky et al., 1997; Varban et al., 1998
	Vitamin E	Reboul et al., 2006
	Carotenoids	During et al., 2005
	Silica	Tsugita et al., 2017
CD36	HDL	Brundert et al., 2011
	LDL	Calvo et al., 1998
	VLDL	Calvo et al., 1998
	Anionic phospholipids	Rigotti et al., 1995
	Apoptotic bodies	Savill et al., 1991
	Collagen	Tandon et al., 1989a
	Aldehyde modified proteins	Duryee et al., 2005
SR-E1/LOX-1	Oxidized LDL	Chen et al., 2001b
	Apoptotic bodies	Li and Mehta, 2000
	C-reactive protein	Shih et al., 2009
	Bacteria	Chen et al., 2001a
	Platelets	Oka et al., 1998
	Anionic phospholipids	Oka et al., 1998
	MAA-Alb	Duryee et al., 2005
SR-H1/STABILIN-1	SPARC	Kzhyshkowska et al., 2006b
	Heparin	Pempe et al., 2012
	oxLDL	Li et al., 2011
	Advanced glycation end-products	Li R. et al., 2009
	Phosphatidylserine	Park et al., 2009
	Phosphorothioate antisense oligonucleotides	Miller et al., 2016
	Placental lactogen	Kzhyshkowska et al., 2008
	GDF-15	Schledzewski et al., 2011
SR-H2/STABILIN 2/HARE	Hyaluronan	Yannariello-Brown et al., 1997
	PINP	McCourt et al., 1999
	Heparin	Harris et al., 2008
	Chondroitin sulfates A-E	Harris et al., 2004; Harris and Weigel, 2008
	oxLDL	Li et al., 2011
	Phosphorothioate antisense oligonucleotides	Miller et al., 2016
	Phosphatidylserine	Park et al., 2008
	Advanced glycation end-products	Li R. et al., 2009; Qian et al., 2009
	VWF-FVIII	Swystun et al., 2018
	Acetylated LDL	Harris and Weigel, 2008
	GDF-15	Schledzewski et al., 2011
SR-E3/MANNOSE RECEPTOR/(CD206)	GalNAc-4-sulfate	Fiete et al., 1998
	Chondroitin sulfates A and B	Fiete et al., 1998
	Terminal mannose	Ezekowitz et al., 1990
	Terminal L-fucose	Taylor et al., 1992
	Terminal GlcNAc	Ezekowitz et al., 1990
	Lysosomal hydrolases	Stahl et al., 1976; Elvevold et al., 2008a
	Tissue plasminogen activator	Smedsrod et al., 1988a
	Procollagen C-terminal propeptides	Smedsrod et al., 1990
	Collagen alpha chains/denatured collagen	Napper et al., 2006; Malovic et al., 2007
	Bacterial and yeast pathogens	Stahl and Ezekowitz, 1998; Gordon, 2002; Allavena et al., 2004
	Influenza, herpes simplex, HIV	Milone and Fitzgerald-Bocarsly, 1998; Reading et al., 2000; Turville et al., 2002
SR-L/LRP-1	ApoE	Hussain et al., 1999
	Tissue plasminogen activator	Salama et al., 2019
	Receptor associated protein (RAP)	Prasad et al., 2016
	α2M, lactoferrin, factor VIIII, etc.	Herz and Strickland, 2001
LSECTIN/CLEC4G	Mannose oligosaccharides	Feinberg et al., 2001
	Terminal GlcNAc, mannose, fucose	Liu et al., 2004
L-SIGN/CD299/CLEC4M	HIV	Boily-Larouche et al., 2012
	SARS-CoV	Jeffers et al., 2004
	HCV	Gardner et al., 2003
	vWF-FVIII	Swystun et al., 2019
LYVE-1	Hyaluronan	Banerji et al., 1999

Kjeken et al. (2001) showed that a clathrin-dependent mechanism is used by LSECs for fluid phase endocytosis. Furthermore, they found that LSECs have higher expression of clathrin protein and twice the number of clathrin-coated pits as compared to KC or hepatocytes. Several years later, Falkowska-Hansen et al. (2007) reported an interesting finding about clathrin-coated vesicles in primary rat LSECs. They found that clathrin heavy chain (CHC) is distributed as net-like structure which was unique to primary rat LSECs. They also showed the co-localization of CHC with microtubules. Furthermore, they found that clathrin-coated vesicle (CCV) function is dependent on microtubules as disruption of microtubules resulted in dysregulation of intracellular transport as well as aberrant signaling of organelles involved in clathrin-mediated endocytosis in LSECs (Falkowska-Hansen et al., 2007). Experiments evaluating the endocytosis capacity of primary LSECs *in vitro* must be performed the same day or within 24 h of purification of the LSECs. After this time period, endocytosis sharply decreases and ceases altogether around day 4 (Braet et al., 1994). Interestingly, the disappearance of fenestrae takes place over the same time course (Braet et al., 2005). To date, the record holder for maintaining LSECs in culture with their native endocytic capacity is the Smedsrod group using an animal-free medium which allowed endocytosis to occur out to 30 days using a pig model (Elvevold et al., 2005). It should be kept in mind that the study of endocytosis in LSECs must be performed with the freshly purified primary cells as no cell line to date expresses all of the specialized receptors nor morphological features that make these cells so dynamic.

## Recently Established Nomenclature for Scavenger Receptors

**FIGURE 2:**
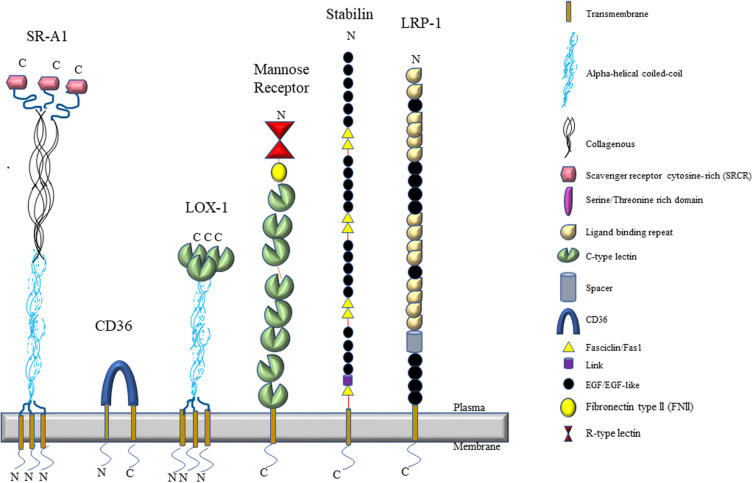
An illustration of the topological features of scavenger receptors expressed by LSECs.

Scavenger receptors are defined as protein receptors that bind to a broad range of ligands from exogenous sources (bacteria, yeast, viruses) and modified endogenous sources [oxidized lipoprotein, advanced glycation end (AGE) products, etc.]. In 2017, a workshop was organized by the National Institute of Allergy and Infectious Diseases at the National Institutes of Health in the United States to develop a clear definition of the various groups of scavenger receptors known to date. This group categorized the scavenger receptors in 11 different classes (A through L) depending on their structure and function and clarified the nomenclature of the various receptors (PrabhuDas et al., 2017). This review will only discuss the role and function of scavenger receptors in the liver sinusoids. LSECs are known to express SR-A, two SR-B variants (SR-B1 and SR-B2/CD36), mannose receptor/CD206/SR-E3, SR-H1 and SR-H2, FcγRIIb and others under normal conditions (Table 1 and Figure 2). LSECs are also known to express SR-E1 under various pathological conditions. Here, an overview of LSEC scavenger receptors are presented.

### Scavenger Receptor A (SR-A)

The class A scavenger receptors (also known as MSR1, SR-AI, SCARA1) were first cloned by the Kreiger group (Kodama et al., 1990; Rohrer et al., 1990) and may have been involved with binding to modified low-density lipoproteins. Scavenger receptor-A (SR-A) is a type II trimeric integral plasma membrane receptor that is encoded on chromosome 8 in both humans and mice. It is characterized by the following six domains: a transmembrane region, a spacer region, a coiled-coil region, a collagenous stretch of repeated Gly-X-P/K, an N-terminal cytoplasmic tail, and a carboxyl terminal-type specific-domain (Figure 2). Alternative splicing results in three variants of SR-A, namely SR-A1.1 and SR-A1.2 (Emi et al., 1993). The structure of the three isoforms are very similar and the differences lie in their cysteine-rich carboxy terminal domain which is involved in cell adhesion and, possibly, bacterial binding (Bowdish and Gordon, 2009; Yap et al., 2015). SR-A type-I is characterized with a cysteine rich carboxy terminal domain whereas SR-A type-II has short C-terminal domain lacking cysteine-rich site at the C-terminus (Rohrer et al., 1990). SR-A type-III has a truncated cysteine-rich domain (Gough et al., 1999). SR-A type1/1.1 are involved in the binding of diverse macromolecules, such as acetylated and oxidized low density lipoprotein (ac/oxLDL) (Suzuki et al., 1997; Kunjathoor et al., 2002), β-amyloid fibrils (El Khoury et al., 1996), AGE products (Araki et al., 1995), molecules present on the surface of gram negative and positive bacteria, such as lipopolysaccharide (LPS) and lipoteichoic acid (LTA) (Dunne et al., 1994). Rohrer et al. (1990) demonstrated that the fibrous coiled-coil and collagen-like domains are responsible for binding different ligands in SR-A type-1/1.1 as removal of cysteine-rich C-terminal domain in SR-A type-II did not affect its binding capacity. SR-A type-1.2 does not bind to SR-A type-1/1.1 ligands but it has been shown to negatively regulate the functions of SR-A type-1/1.1 *in vitro* (Gough et al., 1999).

SR-A expression was once thought to be only expressed in macrophages/KCs, but recent work have shown their expression in brain microglia and astrocytes, LSECs and vascular smooth muscle cells (Nagelkerke et al., 1983; Hughes et al., 1995; Christie et al., 1996; Gough et al., 1999; Godoy et al., 2012). Work conducted in rats have shown that SR-A expressed on LSECs are primarily responsible for carrying out the uptake of the artificial ligand, acLDL, in liver (Nagelkerke et al., 1983). They also theorized the role of an acLDL receptor present on LSECs in the prevention of accumulation of cholesterol under normal condition, though the pathways involved remain elusive. Similarly, another report has demonstrated the uptake of ac/oxLDL by two scavenger receptors; an unidentified 95 kDa protein and the likely candidates, SR-A1/1.1 present on rat LSECs and KCs (De Rijke et al., 1992, 1994; De Rijke and Van Berkel, 1994). In 1990, a commentary in Nature written by Brown and Goldstein had attempted to bring about a universal idea or theory involving the numerous ligands including LDLs that bind to a few scavenger receptors, of which SR-A was a very likely candidate (Brown and Goldstein, 1990). Furthermore, several studies have shown the elevated expression of SR-A in atherosclerotic lesions (Matsumoto et al., 1990) and accumulation of oxLDL, a SR-A ligand in plaques, suggesting it’s putative role in atherogenesis (Hiltunen et al., 2001). SR-AI in LSEC is also involved in uptake and degradation of malondialdehydes-acetaldehyde-serum albumin (MAA), which is considered immunogenic and capable of producing inflammatory responses in the liver (Duryee et al., 2005). It has also been observed that chronic ethanol administration diminishes the uptake of MAA by SR-A in rats. This leads to the accumulation of acetaldehyde and aldehyde modified protein adducts in the circulation. These adducts cause secretion of monocyte chemoattractant protein (MCP)-1 and macrophage inflammatory protein (MIP)-2 via hepatic stellate cells, contributing to alcoholic liver disease (Kharbanda et al., 2001).

Despite all of the evidence that SR-A receptors were involved with the uptake and catabolism of LDLs, the use of knock-out mice in the late 1990’s demonstrated that the SR-A receptors had a negligible effect on liver uptake and decay of serum acLDL (Ling et al., 1997; Van Berkel et al., 1998). Furthermore, SR-A1/1.1-deficient and wild type (WT) mice were compared with the liver sequestration of other non-parenchymal ligands, namely AGE, N-terminal propeptide of type III procollagen (PIIINP) and formaldehyde-treated serum albumin. The results indicated that the SR-A receptors were of minor importance for plasma clearance of these ligands and the distribution in other tissues and organs was not altered (Hansen et al., 2002).

### Scavenger Receptor B (SR-B)

Scavenger receptor class B type 1 (SR-B1/SCARB1) is a member of scavenger receptor B, which is located on chromosome 12 in humans and chromosome 5 in mice (Acton et al., 1994). SR-B1 is a membrane glycoprotein consisting of two short cytoplasmic N- and C-terminal domains, two short hydrophobic membrane regions, and a highly N-glycosylated large looped extracellular domain (Krieger, 1999). They exist as two isoforms, SR-B1 and-B1.1, resulting from alternative splicing (Webb et al., 1997). CD36 and LIMPII Analogous (CLA-1) is the human homolog of SR-B1 (Calvo and Vega, 1993). Liver and steroidogenic tissues (adrenal glands, ovaries, and testis) have been shown to highly express SR-B1 (Acton et al., 1996). Malerod et al. (2002) were the first group to demonstrate the expression of SR-B1 in LSECs from isolated rat liver. Several year later, Ganesan et al. (2016) showed the abundant expression of SR-B1 in mouse LSECs by confocal microscopy. Mutational and knock-out studies have suggested an important role of the SR-B1 receptor in facilitating high density lipoprotein (HDL) uptake in liver and steroidogenic tissues, thereby, its role in atherosclerosis regulation (Acton et al., 1996; Kozarsky et al., 1997; Varban et al., 1998). Other than HDL, SR-B1 also binds to oxLDL, apoptotic cells, unmodified LDL, VLDL (Kozarsky et al., 1997) as well as vitamin E (Reboul et al., 2006), carotenoids (During et al., 2005), and silica (Tsugita et al., 2017). The liver is the primary organ involved in LPS clearance and LSECs are a major contributor for this activity. SR-B1 may be involved with some of the LPS clearance, but as of this date, that is not very clear. There may be a multitude of receptors or “built-in redundancy” in this LPS clearance system so that LPS levels from the gut never get high enough to cause acute inflammation (Ganesan et al., 2016).

CD36 is another member of the Scavenger Receptor B family, which is located on chromosome 7 and chromosome 5 in humans and mice, respectively (Cao et al., 1997). The entire extracellular domain of CD36 and SR-BI share high sequence homology, though the difference lies in their transmembrane and cytoplasmic domain sequences (Acton et al., 1996). It is expressed on adipocytes, capillary endothelial cells, heart and skeletal muscles, and platelets (Talle et al., 1983; Tandon et al., 1989b; Abumrad et al., 1993; Greenwalt et al., 1995; Febbraio et al., 2001) and abundantly expressed on LSECs in the liver (Strauss et al., 2017). The role of CD36 in the metabolism of lipoprotein is well-documented (Febbraio and Silverstein, 2007; Jay and Hamilton, 2018). One study demonstrated the role of CD36 in the uptake of HDL by hepatic NPCs, and their CD36 knock-out mouse model resulted in a modest but significant decrease in the HDL uptake in both hepatocytes and NPCs (Brundert et al., 2011). Similar to SR-B1, CD36 also binds to LDL, VLDL (Calvo et al., 1998), apoptotic cells (Savill et al., 1991) as well as anionic phospholipids (Rigotti et al., 1995), and collagen (Tandon et al., 1989a). Aldehyde modified proteins are likely taken up by CD36 as the SR-A KO mice had significant, but decreased uptake of these proteins (Duryee et al., 2005). An earlier study showed the involvement of CD36 in the endocytosis and degradation of AGE products, implicating its role in diabetes (Ohgami et al., 2001). However, Nakajou et al. (2005) demonstrated that CD36 is not involved in the endocytosis of AGE proteins in LSECs, suggesting the involvement of other scavenger receptors. This is quite plausible since “AGE” is a catch-all for all glycation modifications that occur involving reducing sugars and independent laboratories likely make and use their own “AGE modified protein” preparations.

Upregulation of SR-B1 expression has been documented in the livers of mice fed with a high-fat diet as compared to control mice, suggesting crucial involvement of SR-B1 in NAFLD pathogenesis (Qiu et al., 2013). Translating that to humans, a similar higher SR-B1 expression was observed in type 2 diabetic patients, but no change in hepatic SR-B1 was observed in NASH and hypercholesterolemia patients, predicting only a minimal role of SR-B1 in NAFLD (Rein-Fischboeck et al., 2015). Patients manifesting severe alcoholic hepatitis, inflammation and oxidative stress symptoms had a correlation with a decrease of circulating paroxanase/arylesterase 1 (PON1) in the blood and stimulated alternatively activated (M2) macrophages through activation of CD36. The idea here is that activated macrophages internalize increased amounts of oxLDLs via CD36 and SRA1 and that contributes to the pathophysiology of severe alcoholic hepatitis (Maras et al., 2019). However, none of the studies have documented the contribution of SR-B1 or CD36 expressed on LSECs in maintaining a homeostatic environment in the liver or a modulation in their expression in various diseases.

### Scavenger Receptor E (SR-E)

SR-E1/LOX-1, also known as lectin-type oxidized LDL receptor or oxidized LDL receptor 1 (OLR1) is a type II transmembrane protein belonging to scavenger receptor E family. It is located on chromosome 12 in humans and chromosome 6 in mice. It consists of a transmembrane domain, a short cytoplasmic domain at the N-terminus, a connecting neck domain and a C-type lectin-like extracellular domain at the C-terminus (Sawamura et al., 1997; Park et al., 2005). Maturation of Lox-1 requires post-translational N-linked glycosylation of the extracellular domain at the C-terminus and this modification was shown to be important for binding of various ligands, intracellular transport, signaling processes, and several other biological functions (Kataoka et al., 2000). Mutational studies have shown the importance of conserved C-terminal residues in the lectin like domain in LOX-1 for ligand binding (Chen et al., 2001b). LOX-1 is expressed by endothelial cells, macrophages, vascular smooth muscle cells, adipocytes and chondrocytes in low levels (Sawamura et al., 1997; Yoshida et al., 1998; Chui et al., 2005; Akagi et al., 2006). However, an increase in expression is seen by various pro-inflammatory markers, shear stress and mechanical stimuli, such as tumor necrosis factor-alpha (TNF-α), and phorbol 12-myristate 13-acetate (PMA) *in vitro* (Kume et al., 1998; Murase et al., 1998). Despite having no homology with other oxLDL binding SRs, such as SR-A and B, it is considered as the major endothelial SR for binding, uptake, and degradation of oxLDL (Sawamura et al., 1997). Other than oxLDL, LOX-1 is also involved in the binding with apoptotic and aged cells, C-reactive protein (CRP), bacteria, platelets and anionic phospholipids, such as phosphatidylserine and phosphatidylinositol, suggesting their role in various physiological conditions (Oka et al., 1998; Li and Mehta, 2000; Chen et al., 2001a; Shih et al., 2009).

Under normal conditions, LOX-1 is expressed in low amounts, in contrast to its high expression during various pathophysiological events (Ogura et al., 2009; Balzan and Lubrano, 2018). A study has documented an increased expression of LOX-1 in the aorta of hypercholesterolemic, hyperlipidemic rabbits suggesting that a Western diet may induce this receptor for the overall metabolism of lipids (Chen H. et al., 2000; Chen M. et al., 2000). Another study was conducted on a hypertensive rat model using Dahl salt-sensitive and Dahl salt-resistant rats. Dahl salt sensitive rats develop hypertension when fed with a high salt diet and Dahl salt resistant rats show minor blood pressure changes on the same diet. They reported up-regulation of LOX-1 expression in hypertensive rats, thereby, suggesting its role in hypertension and a putative target for treating atherosclerosis (Nagase et al., 1997, 1998). The role of LOX-1 contributes to the pathology NAFLD resulting, in part, from LSEC dysfunction. Zhang et al. (2014) studied the effect of human LSEC LOX-1 gene knockdown in oxLDL-induced hepatic injury. They found an increase in ROS production and enhanced p65 expression in oxLDL treated human LSECs. This effect was significantly reduced after LOX-1 siRNA treatment, suggesting the role of LOX-1 in activation of NF-kB and the ROS pathway. A reduction in the expression of eNOS was found in human LSEC culture treated with oxLDL, whereas, LOX-1 siRNA treatment resulted in an enhanced expression of eNOS in this culture system. They also reported a reduction in the number of fenestrae, diameter and porosity in ox-LDL-treated human LSEC culture and LOX-1 siRNA resulted in reversal of these effects, suggesting its role in stress related atherogenesis (Fraser et al., 1995). LSEC defenestration was found to be mediated by LOX-1 by upregulating ET-1 and caveolin-1. Although, the association between LOX-1, ET-1, and caveolin-1 remains unclear, oxidative stress generated due to ROS production suggests the role of LOX-1 in NASH and NAFLD pathological manifestations (Pasarin et al., 2012). Along with SR-A, LOX-1 was also shown to be responsible for degradation of MAA-Alb (Duryee et al., 2005). Furthermore, a study conducted on aortic endothelial cells reported a reduction in LOX-1 expression with aging and associated it with the progression of aging related diseases (Khaidakov et al., 2011).

Mannose receptor (MR)/CD206/SR-E3 is another member of the scavenger receptor E belonging to the C-type lectin family. It is a type I integral membrane protein which is present on chromosome 10 in humans and chromosome 2 in mice. The mannose receptor is composed of an N-terminal extracellular region and a C-terminal intracellular region. The N-terminus is composed of three distinct domains and each domain binds to its specific ligands. First, eight consecutive C-type carbohydrate recognition domains (CRDs) that bind to terminal mannose residues, N-acetylglucosamine and L-fucose (Ezekowitz et al., 1990; Taylor et al., 1992). Second, a fibronectin type-II repeat domain that binds to collagen I-IV (Martinez-Pomares et al., 2006; Napper et al., 2006). Third, a cysteine-rich domain at the N-terminus binds to sulfated sugars, such as GalNAc-4-sulfate and chondroitin sulfates A and B (Fiete et al., 1998). Besides binding of specific ligands by their respective domains, MR is involved in the binding of numerous endogenous ligands, such as lysosomal hydrolases (Stahl et al., 1976; Elvevold et al., 2008a), tissue plasminogen activator (tPA) (Smedsrod et al., 1988a), and Procollagen type I carboxy-terminal propeptide (PICP) (Smedsrod et al., 1990). MR has been implicated in the binding of several pathogens including *Candida albicans*, *Pneumocystis carinii*, and *Leishmania donovani* by its cysteine-rich domain (Stahl and Ezekowitz, 1998; Gordon, 2002; Allavena et al., 2004). More recent studies have reported MRs binding with influenza, herpes simplex virus as well as HIV (Milone and Fitzgerald-Bocarsly, 1998; Reading et al., 2000; Turville et al., 2002) with varying affinities. The MR is expressed in most tissue macrophages, LSECs, lymph node and spleen; kidney mesangial cells, and dendritic cells subsets (Linehan et al., 1999; McGreal et al., 2004; Linehan et al., 2005).

Due to their ability to recognize and bind with carbohydrate moieties present on the surface of pathogens, MR expressed on LSECs is involved in the clearance of denatured collagen (Malovic et al., 2007). This study showed a reduction in plasma clearance of radiolabeled DebColl, a heat-denatured type-1 collagen, in a MR KO mouse model *in vivo*. LSECs isolated from MR KO mice were not able to internalize radiolabeled DebColl as efficiently as WT LSECs *in vitro*. Reduction in the endocytic capacity of LSECs for removing denatured collagen might contribute to the onset of pseudo-capillarization or fibrosis (Ala-Kokko et al., 1987). Subsequently, Elvevold et al. (2008a) reported the importance of MR in maintaining the high lysosomal degradation capacity using the MR knock-out model. They found a reduction in lysosomal enzymes in freshly isolated LSECs from MR KO mice as compared to WT. They further examined the endocytic and intracellular degradative capacity of LSECs and reported that MR KO mice took twice as long to degrade injected radiolabeled formaldehyde-treated serum albumin (FSA) as compared to WT, though endocytic capacity remained the same for both cell types. In accordance with the former, the MR KO mice contained nearly twice as much radioactivity than the WT mice 2 h-post-injection. It was concluded that the cellular uptake of SR ligand is not hampered in MR KO mice and there is only a deficiency in the amount of lysosomal enzyme content, hence, reduced degradative capacity in MR KO mice. This demonstrates that the MR is involved with the turnover and homeostasis of many intrinsic molecules within the mammalian organism.

Several studies have shown the expression of MR on LSEC is impacted by cytokines and inflammatory stimuli. Asumendi et al. (1996) demonstrated the upregulation of the LSEC MR after exogenous administration of human recombinant IL-1b in the rat *in vivo*. IL-1b is a pro-inflammatory cytokine present in acute infections. A similar upregulation was observed in the LSEC MR when IL-1b was induced with LPS in the rat endogenously (Asumendi et al., 1996). Similarly, enhanced MR expression by LSECs in culture when incubated with IL-10 or IL-4 with IL-13 (Liu Y. et al., 2013) acts contrary to IL-1b and is involved with the “type-II” activation of the immune system. Similarly, a study on mouse model of C26 colon carcinoma hepatic metastasis has shown that MR expression level and endocytic capacity increases with an increase in the production of IL-1b from LSECs. The increased production of IL-1b resulted from the binding of ICAM-1 expressed on LSECs with LFA-1 on C26 colon carcinoma cells (Arteta et al., 2010). This activity decreased secretion of IFN-γ as well as anti-tumor cytotoxicity. This study discovered the role of MR in promoting IL-1b and ICAM-1 mediated pro-metastatic effects in the liver.

### Scavenger Receptor H (SR-H)

Stabilin (or FEEL/CLEVER/HARE) receptors are class H scavenger receptors. They are type I transmembrane proteins consisting of 20–21 EGF/EGF-like domains, seven fasciclin-1 domains, an X-linked domain, a transmembrane region, and a short cytoplasmic domain (Politz et al., 2002). This family consists of two members; Stabilin-1 and Stabilin-2. Stabilin-1 is also known as SR-H1/MS-1/FEEL-1/CLEVER-1. Stabilin-2 is also known as SR-H2/FEEL-2 and HARE, which is a shorter isoform of Stabilin-2 generated by proteolytic cleavage (Goerdt et al., 1991; Weigel, 2019). The main structural difference between Stabilin-1 and Stabilin-2 is the presence of 20 EGF-like domains in stabilin-2 as compared to 21 domains in stabilin-1. Stabilin-1 is located on chromosome 3 and chromosome 14 in humans and mice, respectively. Stabilin-2 is located on chromosome 12 in humans and chromosome 10 in mice, respectively. Their extracellular domains share 55% similar homology, but their short intracellular domains are highly diverse, which results in differential abundance in different tissues and cells (Harris and Cabral, 2019). Sinusoidal endothelial cells in the liver, spleen, adrenal cortex and tissue macrophages express Stabilin-1 (Goerdt et al., 1991; Kzhyshkowska et al., 2006a), whereas, the expression of stabilin-2 is abundant in the sinusoids of liver, spleen, lymph node and bone marrow (Yannariello-Brown et al., 1997; Weigel and Weigel, 2003; Qian et al., 2009). Both receptors are also expressed in several other tissues throughout the body at lower levels (Falkowski et al., 2003). Stabilin-1/2 are considered the primary scavenger receptors of LSECs and are responsible for the binding, uptake, and degradation of multifarious ligands, such as hyaluronan, N-terminal pro-peptide of type I procollagen (PINP) (Yannariello-Brown et al., 1997; McCourt et al., 1999), chondroitin sulfates (Harris et al., 2004), AGE (Li R. et al., 2009), oxLDLs (Li et al., 2011), SPARC (Kzhyshkowska et al., 2006b), heparin (Harris et al., 2008), von Willebrand factor-factor VIII (Swystun et al., 2018) and synthetic phosphorothioate antisense oligonucleotides (Miller et al., 2016).

Stabilin-1, and to some extent, Stabilin-2, in LSECs, are specifically involved in the uptake of oxLDL, thus playing a role in the prevention of atherogenesis (Li et al., 2011). The Stabilin receptors are also involved in the internalization and clearance of various macromolecules that cannot be cleared by the kidney due to size limitation for some of these macromolecules. In 2011, it was reported that mice lacking both Stabilin-1 and Stabilin-2 did not live as long as their WT littermates due to mild perisinusoidal liver fibrosis and severe glomerular fibrosis. This study showed that both Stabilin-1 and -2 are essential for the normal clearance of extracellular matrix material, thus preserving the homeostasis of the liver as well as other distantly located organs (Schledzewski et al., 2011). In humans, it is not known what occurs in Stabilin allelic insufficiency or loss of function. However, in de-differentiated tissues in cases of liver cancer, loss in the expression of Stabilin-1 and -2 was seen in hepatocellular carcinoma (HCC) patients, and this loss was inversely related to patient survival. Additionally, loss of Stabilin-1 and -2 as well as CD32b was also observed around the tissues surrounding the tumor in HCC patients (Geraud et al., 2013). Conversely, another study has shown that Stabilin-1 retains its expression in the LSECs of the diseased liver and mediates transmigration of T cells, especially T regulatory cells across the endothelium in the inflamed liver. They also found increased expression of Stabilin-1 in the vessels and sinusoids lining the tumor in HCC (Shetty et al., 2011). A survey was conducted on young and old rats to delineate Stabilin expression and endocytic capacity in LSECs in response to aging. Their results found that there was an attenuation in the endocytic capacity of old rats, although the degradation capacity for both ages were similar. An increase in LSEC thickness was also observed in old age rats, which might be responsible for lower endocytic capacity (Simon-Santamaria et al., 2010). Similarly, another study had documented a decrease in Stabilin-2 receptors expression on primary LSECs in an aged rat model (Maeso-Diaz et al., 2018). From these studies, we may generally conclude that aging results in reduced expression of the Stabilin receptors and disease may alter expression and function of these receptors.

LSECs are involved in the synthesis of Factor VIII, a blood coagulation factor. This pro-coagulant is either defective or missing in Hemophilia A patients (Powell, 2009). Do et al. (1999) first identified FVIII mRNA in purified LSECs and hepatocytes from mice and in cultured LSECs by RT-PCR. They also found higher FVIII mRNA expression in LSECs quantitatively. In agreement with this finding, the transplantation of LSECs isolated from FVB/N-Tie2–GFP mice in a hemophilia A mouse model restored plasma level of FVIII (Follenzi et al., 2008). An increase in plasma FVIII correlated with the proliferation of transplanted LSEC in a Hemophilia A mice model. Two months after the LSEC transplantation, a bleeding experiment was performed to evaluate coagulation. The results showed that bleeding stopped in 15–20 min after a tail-cut which corrected this disorder. To take this one step further, an interesting attempt was made to transplant human fetal LSECs in uPA-NOG (or immunodeficient) mice and evaluate human FVIII plasma levels in mice (Fomin et al., 2013). Factor VIII levels were about half as high as a normal human plasma sample, but the amount far surpassed the non-transplanted controls indicating that LSECs produce FVIII in appreciable quantities. These findings suggest a novel role of LSECs in the treatment of hemophilia A patients suffering from FVIII deficiency. Interestingly, Stabilin-2 which is highly expressed in LSECs and in the sinusoids of spleen, also regulates the clearance rates of FVIII. Based on the data derived from genome wide association studies (GWAS), both Stabilin-2 and CLEC4M (see below) have been identified to affect vWF-FVIII levels in plasma. vWF or von Willebrand factor is normally found in tight physical association with FVIII in plasma (Lollar, 1991). Using a combination of KO mice and immunofluorescence techniques, Swystun and co-workers firmly established that Stabilin-2 and CLEC4M expressed in LSECs of mice, regulates turnover of FVIII (Swystun et al., 2018, 2019).

### Scavenger Receptor L (SR-L)

LRP-1 (Low-density lipoprotein receptor-related protein or CD91 or α2macroglobulin receptor) is a scavenger and endocytic receptor present on the cell surface belonging to the family of low-density lipoprotein receptor (Herz et al., 1988). The structure of LRP-1 is composed of five domains (i) the ligand-binding domain (ii) the O-linked sugar domain (iii) the EGF-precursor homology domain (iv) the transmembrane domain and the intracellular domain. Proteolytic cleavage of LRP-1 results in alpha subunit ligand-binding domain at the N-terminus and ß subunit consisting of other remaining domain at the C-terminus (Lillis et al., 2008). LRP-1 binds to more than 30 ligands, such as ApoE (Hussain et al., 1999), tPA (Salama et al., 2019), a receptor-associated protein (RAP) (Prasad et al., 2016), in addition to trypsin-activated α2-macroglobulin (α2M^∗^), lactoferrin, Factor VIII and others (for a complete list of ligands, see Herz and Strickland, 2001). The presence of different motifs at the cytoplasmic tail of LRP-1, such as two dileucine motifs, NPXY motifs, one YXXL motif has been suggested to be responsible for its high endocytic uptake (Li et al., 2000; Deane et al., 2008). LRP-1 is expressed in the liver, lung, brain, intestine, and muscles (Herz et al., 1988).

Oie et al. (2011) reported the expression of LRP-1 on LSECs with immunofluorescence. They showed that LRP-1 expressed in LSECs is responsible for partial hepatic clearance of RAP, an inhibitor of all known ligand interactions with LRP-1 and α2M^∗^, along with other hepatic cells, suggesting the role of LSECs in lipid homeostasis. A hepatocyte-specific LRP-1 KO study showed that NAFLD disease progression is accelerated by the deficiency of LRP-1 in mice fed with a high-fat high cholesterol diet and that LSEC and KC LRP-1 were not sufficient to rescue the phenotype (Hamlin et al., 2018). Similarly, another hepatocyte-specific LRP-1 KO study has demonstrated an increase in the accumulation of lipid in primary culture of LRP-1 KO hepatocytes due to an impairment in the lysosomal degradation capacity of auto-phagolysosomes leading to cell death (Hamlin et al., 2016). These studies suggests severe impact of LRP-1 dysregulation on NAFLD progression. However, not much is known about the LSEC specific function of LRP-1 in various diseases and this needs to be elucidated.

## Other Cell Surface Receptors

### FcγRIIb/CD32b

Fc gamma receptors have four major classes in mice (FcγRI-IV) and six in humans (Daeron, 1997). Fc gamma receptors IIb (FcγRIIb) binds to the monomeric immunoglobulin G (IgG) Fc domain with low affinity and inhibit the response produced by activating FcγRs. It is located on chromosome 1 in both humans and mice (Fagerberg et al., 2014; Yue et al., 2014). FcγRIIb consists of a cytoplasmic region characterized by a 13 amino acid long YSLL sequence, also known as Immunoreceptor Tyrosine based Inhibitory motif (ITIM) and an extracellular domain (Van den Herik-Oudijk et al., 1995). Alternative splicing of mRNA sequence results in two isoform namely FcγRIIb1 and FcγRIIb2. ITIM is essential for its inhibitory function (Van den Herik-Oudijk et al., 1995). When immunoglobulins on the cell surface bind and crosslink these receptors, tyrosine 309 is phosphorylated. Tatsushi et al. demonstrated that mutation of Tyr 309 to phenylalanine in the ITIM motif abolished its inhibitory effect of B cell activation (Amigorena et al., 1992; Muta et al., 1994). Daeron et al. (1993) demonstrated that a single mutation of Tyr26 to glycine at the intra-cytoplasmic domain and YSLL motif resulted in impairment of endocytosis and phagocytosis processes, suggesting that phosphorylation or dephosphorylation of tyrosine residues is important for normal function of the receptor. Similarly, another study reported that single amino acid substitution of tyrosine to alanine using alanine scanning mutagenesis methods resulted in the abolition of endocytosis (Hunziker and Fumey, 1994). This investigation discovered that the endocytosis signaling and di-leucine-based signaling overlap so that you could not have one without the other in terms of function. The expression of FcγRIIb is found on the sinusoids of spleen and liver as well as in immune cells, such as B cells, dendritic cells, myeloid cells as well as leukemia and lymphoma cells (Lim et al., 2011; Tutt et al., 2015).

FcγRIIb has been implicated in the regulation of immunoreactivity. One study has reported a decrease in expression of FcγRIIb on B cells in active systemic lupus erythematosus (SLE) patients as compared to healthy control, suggesting its protective role in SLE (Ochi and Kawabi, 1992). Similarly, an increase in FcγRIIb expression on B cells upon delivery of retroviral transduced FcγRIIb bone in spontaneous lupus-prone mice compared to the mice that received parent retrovirus transduced bone marrow (McGaha et al., 2005). They also found a decrease in the immune complex accumulation in the kidney as compared to control. Besides its expression on hematopoietic cells, FcγRIIb expression is also found exclusively on LSECs, and it is used as a marker to distinguish LSECs from other liver cell types (Mousavi et al., 2007). In hepatic sinusoids, FcγRIIb is responsible for removing small immune complexes (*SIC*). Ganesan et al. (2012) studied the blood clearance rate of *SIC* using radio iodinated *SIC* in WT and FcγRIIb KO mice model. They found an inhibition in the clearance rate of *SIC* in FcγRIIb KO mice as compared to control, suggesting the involvement of LSECs FcγRIIb in immune complex mediated diseases (Ganesan et al., 2012).

A study conducted on NAFL and NASH biopsy specimens to assess the expression of FcγRIIb on LSECs reported a medium negative correlation between serum collagen type IV and hyaluronan with FcγRIIb expression (Ishikawa et al., 2019). An increase in type IV collagen and hyaluronan contents have already been shown with NASH progression, suggesting a reduction in scavenger function of FcγRIIb. They also witnessed an inversely proportional relation between FcγRIIb expression and fibrosis stages, reporting the highest expression at the initial stage of fibrosis and lowest at fibrosis stage 3. However, taking all of the data together across the various grades of fibrosis and NAFLD activity scores, Ishikawa and coworkers did not find a significant difference in expression level of FcγRIIb. A new insight in the hepatic fibrosis study was provided by Vilar-Gomez et al. (2017) who reported platelet counts as a novel indirect biomarker for portal hypertension and advanced hepatic liver disease assessment. They reported the inhibitory role of platelets in fibrosis. In agreement with this study, Ishikawa et al. (2019) have shown a positive relationship between platelet count and FcγRIIb expression on LSECs through regression analysis, suggesting a low platelet count in high fibrosis stage, thereby, decreased FcγRIIb expression. For HCC, a decrease in FcγRIIb expression is co-commitment with an increase in cancer grade similar to Stabilin-2 (Geraud et al., 2013). A study conducted on peritumoral tissue samples of HCC patients showed that the expression of FcγRIIb was decreased in 63% of samples taken into consideration for the study. A microarray analysis of these tissues found that loss of FcγRIIb is related to significantly longer tumor-specific survival. However, in terms of the 5-yr survival rate, there was more of an impact or survival rate for Stabilin-2 (42%) than FcγRIIb (16%).

Another ligand of FcγRIIb, fibrinogen-like protein 2 (FGL2), was found to be increased in NAFLD patients demonstrating severe forms of NAFLD, suggesting a decrease in FcγRIIb expression (Colak et al., 2011). This correlation did not hold across the fibrosis stages or grades of steatosis. Furthermore, Maeso-Diaz et al. (2018) have shown an even or slight increase in FcγRIIb expression in the comparison of young and old LSECs, however, there is a very stark reduction in Stabilin-2, eNOS, BMP-2, Lamb1, and HGF in aged LSECs suggesting that the LSECs become vulnerable to acute or chronic injury in old age.

### Toll-Like Receptors

Besides scavenger receptors, LSECs express multifarious pattern recognition receptors mostly consisting of toll-like receptors (TLRs). TLRs are capable of recognizing pathogen-associated molecular patterns (PAMP) present on invading microbes or damage-associated molecular pattern (DAMP) originating from endogenous damaged or apoptotic cells (Kawasaki and Kawai, 2014). They are type I transmembrane glycoprotein consisting of an extracellular N-terminal ligand-binding domain, single transmembrane domain, and a C-terminal cytoplasmic domain. Ligand binding is mediated through an ectodomain characterized by leucine-rich repeats (Bell et al., 2003). Downstream signaling is mediated by adaptor proteins associated with the Toll/IL-1 receptor (TIR) present at the cytoplasmic C-terminal domain (O’Neill and Bowie, 2007). TLRs form an important bridge between innate and adaptive immune response system (Werling and Jungi, 2003; Pasare and Medzhitov, 2004). TLRs also induce the production of pro-inflammatory and effector cytokines and aid in the activation of T-cells by upregulating co-stimulatory molecules present on antigen-presenting cells (Vasselon and Detmers, 2002). Humans express 10 TLRs (TLR 1–10), whereas, mice express 12 TLRs (Hopkins and Sriskandan, 2005). Of these, LSECs express seven Toll-like receptors; TLR1–4, 6, 8, 9.

Uhrig et al. (2005) demonstrated a constitutive expression of TLR4 on cultured LSECs isolated from mice. TLR4 binds to LPS present on gram-negative bacteria and initiates an immune response for its clearance (Poltorak et al., 1998). In this study, cultured LSECs developed tolerance upon repetitive exposure with LPS, though this effect was not mediated by downregulation in the expression of TLR4. Reduction in the activation of NF-kB was responsible for developing tolerance against LPS stimulation in LSECs. Not too long thereafter, Martin-Armas et al. (2006) showed that CpGs are taken up by TLR9 present in murine LSECs. The bacterial DNA is characterized by unmethylated CpG motifs that act as a potent immune stimulator by inducing the production of cytokines from various immune cells, such as dendritic cells, macrophages, B cells and NK cells (Ashkar and Rosenthal, 2002; Dalpke et al., 2006). These studies reported the presence of TLR9 in murine LSECs for the first-time using RT-PCR and immunolabeling. LSECs accumulate the most FITC-labeled CpG than other liver cell types shown by both *in vitro* and *in vivo* experiments. They also reported the binding of CpG to TLR9 in the endo-lysosomal compartment which activated NF-κB signaling for the IL-1β and IL-6 production. This study suggests the role of LSECs in mediating innate immune response in the liver. A TLR4 KO mouse study demonstrated the role of TLR4 in NAFLD by enhancing the secretion of hepatic TGF-ß and collagen associated with fibrosis (Sutter et al., 2016). These results strongly suggest that the chronic inflammation associated with fatty liver disease is regulated, in part, by TLR4.

What is the functional role of TLRs in response to ligands in LSECs? To answer this question, responses to many TLR specific agonists were evaluated in murine LSECs (Wu et al., 2010). Upon treatment with TLR specific agonists, the high mRNA expression level of TLR1,4,6,8 and moderate mRNA expression level of TLR9 were observed in murine LSEC, however, TLR5, 6, and TLR9 showed very low mRNA expression. Contrary to mRNA expression, TLR5 and TLR6 did not show any expression at the protein level in flow cytometry. They also showed that the antiviral response is produced by TLR3 in LSECs by the secretion of IFN-β. Furthermore, TNF-α production was reported in LSECs treated with TLR4 agonists in high amounts and by TLR2,3 and TLR8 in a moderate amount. Similarly, a 4-fold and 16-fold upregulation was observed in LSECs treated with TLR4 and TLR3 agonists, respectively. LSECs were also able to upregulate MHC class II expression with TLR8 agonists as well as the proliferation of T-cells with TLR1,2 and TLR6 agonists. Additionally, LSECs were involved with the activation of CD4 and CD8 T-cells when treated with TLR1,2 and TLR6 agonists. These results suggest that LSECs are capable of initiating antiviral and pro-inflammatory responses by TLR3 as well as adaptive responses through TLR1,2,6 and TLR8, thereby maintaining hepatic immune response. Similarly, the role of TLR3 stimulated the murine LSECs is reported in suppressing Hepatitis B viral replication mediated by IFN-ß production *in vitro* (Wu et al., 2007).

The portal circulation continuously exposes the liver to microbial antigens and food from the gut; therefore, it possesses a piece of special machinery to maintain immune tolerance. Liu J. et al. (2013) reported that incubation of LSECs with palmitoyl-3-cysteine-serine-lysine-4 (P3C), a TLR2 ligand, resulted in the reversal of immune tolerance. LSECs were co-cultured with stimulated T cells isolated from mice and treated with P3C. This resulted in an increase in proliferation of T cells as well as cytokine production in co-culture as compared to T cells cultured alone. An increase in CD8+ effector T cell population, IL-12 production, and decrease in PD-L1 expression on LSECs was also observed in these co-culture systems. This study outlined the role of TLRs in regulating the immunosuppressive property in LSECs. Similarly, one study has demonstrated the role of TLR2 expressed on LSECs in initiating an innate immune response toward adeno-associated viral vectors (rAAV) and efficiency of gene therapy mediated by rAAV which may be enhanced by understanding this mechanism (Hosel et al., 2012). As the anatomical sieve of the liver, the LSECs are continually monitoring antigens and confer tolerance to maintain proper homeostasis.

### L-SIGN and LSECtin

Liver/lymph node-specific ICAM-3 grabbing non-integrin (L-SIGN)/CD299L/CLEC4M and Liver and lymph node sinusoidal endothelial cell C-type lectin (LSECtin)/CLEC4G are type II transmembrane proteins belonging to the C-type lectin family (Bashirova et al., 2001; Pohlmann et al., 2001; Gardner et al., 2003; Liu et al., 2004). These two receptors are encoded on chromosome 19 in humans (Grimwood et al., 2004). They are characterized by an intracellular domain, a transmembrane domain, and an extracellular domain composed of a neck domain and a C-type carbohydrate recognition domain (CRD). Ligand binding is mediated through C-type CRD. L-SIGN is expressed by liver and lymph node sinusoidal endothelial and placental capillary endothelial cells (Pohlmann et al., 2001). Likewise, liver and lymph node sinusoidal endothelial cells specifically co-express LSECtin (Liu et al., 2004). LSECtin expression is also seen on bone marrow sinusoidal endothelial cells as well as on KCs in the liver (Dominguez-Soto et al., 2009). L-SIGN binds to high mannose oligosaccharide (Feinberg et al., 2001), whereas, LSECtin can bind with N-acetylglucosamine, mannose, and fucose (Liu et al., 2004). Since L-SIGN is expressed on placental capillary endothelium, one study has shown their possible involvement in mother to child transmission of HIV-1 virus (Boily-Larouche et al., 2012), while several other studies relating L-SIGN with HIV-1 entry remain contradictory. L-SIGN expressed on pulmonary endothelial cells serve as the gateway for the entrance of SARS-CoV as it was able to bind HEK293T cells expressing purified soluble SARS-CoV glycoproteins (Jeffers et al., 2004). Similarly, LSECtin might also play a role in mediating SARS-CoV infection in hepatocytes (Gramberg et al., 2005).

L-SIGN has been reported to interact with glycoprotein E2 of the Hepatitis C viron (HCV) (Gardner et al., 2003). This study has shown that L-SIGN transfected Hela cells were able to bind purified HCV-E2 protein as compared to parental HeLa cells using FACS analysis. Since, mannan is a ligand for L-SIGN, incubating the recombinant L-SIGN expressing HeLa cells (HeLa-L-SIGN) with mannan inhibited binding between L-SIGN and purified HCV-E2 protein. They confirmed the finding by exposing the HeLa-L-SIGN cells to HCV-virion and detected the HCV genome in L-SIGN transfected Hela cells by RT-PCR and Southern blotting. Similarly, another group has shown the involvement of L-SIGN in facilitating the entry of HCV and passing it to nearby hepatocytes present in the liver using HCV pseudotype particles (Lozach et al., 2004). Since LSECtin and L-SIGN belongs to the same C-type lectin family and share a 32% sequence identity, Li Y. et al. (2009) demonstrated that the central domain of LSECtin binds with L-SIGN and along with the C terminal CRD domain bind with E2 glycoprotein present on HCV suggesting that LSECtin binding with L-SIGN might play a role in the HCV binding to LSECs. LSECtin is also involved in mediating T-cell immune response in the hepatic system (Tang et al., 2009). This study showed that LSECtin binds to CD44, a hyaluronan binding receptor, present on activated T cells, halting T-cell activation and proliferation, thereby preventing liver injury. Both of these receptors bind to mannose residues, which help them in clearing pathogens from circulation (Liu et al., 2004). These studies suggest that L-SIGN and LSECtin may be targeted for the treatment of HCV and inflammatory liver diseases.

### LYVE-1

Lymphatic vessel endothelial hyaluronan receptor 1 (LYVE-1) is a type I integral membrane glycoprotein which was firstly identified exclusively on lymph vessels responsible for sequestering hyaluronic acid in the lymph vessel endothelium (Banerji et al., 1999). Similar to its homolog CD44, LYVE-1 contains a single Link module in the extracellular domain that is responsible for Hyaluronic acid (HA) binding (Day and Prestwich, 2002). It is present on chromosome 11 in humans and chromosome 7 in mice. With the use of better antibodies, LYVE-1 expression was also detected in the liver, spleen, and lymph node sinusoidal endothelial cells in humans (Mouta Carreira et al., 2001; Akishima et al., 2004). However, a recent study conducted in rodents has revealed their expression in the non-sinusoidal endothelium of many other organs, such as lungs, heart, and adrenal gland (Zheng et al., 2016). Once regarded as the main receptor responsible for the internalization and transport of HA in the lymph circulation, a study using LYVE-1 KO mice model revealed that it is not crucial for the metabolism of HA in the lymphatic endothelium (Gale et al., 2007). Since HA is the only known ligand for LYVE-1, to date, and LSECs play a very important role in the degradation of HA, Mouta Carreira et al. (2001) hypothesized that LYVE-1 expression might be present on LSECs. They performed immunohistochemistry analysis and confirmed the expression of LYVE-1 on human and murine LSECs (Mouta Carreira et al., 2001). Since LSECs are the only LYVE-1 expressing cells in the liver, it can be used to distinguish LSEC from other liver cell types. Earlier studies had suggested a link between increased HA level and cirrhosis (Ichida et al., 1996). This study showed that LSECs from the cirrhotic and HCC liver had a lower capacity to degrade HA and serum levels of HA were increased. We now know that Stabilin-2/SR-H2/HARE previously described in this article is the determining factor for HA degradation in liver (Eriksson et al., 1983; Harris et al., 2007).

Similarly, data from murine as well as a human models of HCC reported a reduction in the LYVE-1 expression in liver tumors with immunohistochemical (IHC) analysis (Geraud et al., 2013). Additionally, a tissue microarray analysis of 191 HCC samples found complete loss of LYVE-1 expression in 83% of the cases as compared to control. There was also a positive correlation between LYVE-1 expression and histological grade of the individual tumor with a 47% loss in grade G1 (least) and 89% in grade G3 (most). Furthermore, Arimoto et al. (2010) conducted an IHC experiment on frozen normal and diseased liver tissue and found decreased expression of LYVE-1 and increased vWF expression in inflamed or fibrotic liver. A weak negative correlation was also observed between LYVE-1 expression and fibrosis stage. An ultrastructural analysis revealed loss in LSEC fenestration and appearance of the basement membrane-like structure in the diseased liver, suggesting the onset of sinusoidal capillarization. They found a similar reduction in LYVE-1 expression in chronic viral hepatitis and virus-related cirrhosis liver tissues. This study suggests the possible role of LYVE-1 in the progression of liver fibrosis. An increase in vWF expression with a similar decrease in LYVE-1 expression poses disturbances in microcirculation. Sinusoidal capillarization results in circulatory problems and disturbs the transport of various macromolecules between blood and hepatocytes in the diseased liver state. Given their role in hepatic disease progression, LYVE-1 and vWF may be used as a potential marker for sinusoidal capillarization.

### Adhesion Molecules Expressed by LSECs

Adhesion molecules play an important role in mobilizing leukocytes at the site of inflammation. This process involves several steps and carried out by a different set of adhesion molecules, such as integrins, the selectins, and Ig superfamily members (Shetty et al., 2018). Each of the adhesion molecules are tightly regulated to maintain a homeostatic environment and their expression is modulated under certain diseased condition. Unlike capillary and microvascular endothelial cells, LSECs express few integrins. Integrins are heterodimers consisting of alpha and beta subunits mediating cell-extracellular matrix adhesion (Bokel and Brown, 2002). Couvelard et al. (1993) evaluated the expression of different cell-matrix adhesion proteins in LSECs and found that LSECs express only α1ß1 and α5ß1 under normal condition whereas, αVß3 and αIIbß3 in low and variable levels. α5ß1 acts as fibronectin receptor (Schaffner et al., 2013), whereas, α1ß1 binds to collagen (Eble et al., 1993). αVβ3 binds to vitronectin (Horton, 1997) as well as fibronectin (Van Agthoven et al., 2014) and αIIbß3 binds to fibrinogen (Wippler et al., 1994). Furthermore, they found a strong enhanced expression of α1ß1 and α5ß1 as well as αVß3 and αIIbß3 in cirrhotic liver that were faintly expressed in normal liver. In addition, other integrins (α6ß1, α6ß4, α2ß1, and α3ß1) that did not show any expression in normal liver, and showed an increased expression in cirrhotic liver, suggesting their contribution to capillarization. These integrins bind to laminin, an important component of the basal lamina of the extracellular matrix (Languino et al., 1989; Felch et al., 1992; Lee et al., 1992; Chang et al., 1995). Several studies have documented an increased deposition of collagen IV, fibronectin and laminin in LSEC basement membranes during liver fibrosis and inflammation (Walsh et al., 2000; Xu et al., 2003; Mak and Mei, 2017), suggesting a possible role of these integrins in inflammation and fibrosis.

Similar to microvascular endothelial cells, the LSEC Ig-superfamily of adhesion molecules is composed of ICAM-1 (Intercellular adhesion molecule-1), ICAM-2 (Intercellular adhesion molecule-2), VCAM-1 (Vascular cell-adhesion molecule) and PECAM-1 (Platelet endothelial cell adhesion molecule/CD31). VCAM-1 is an adhesion molecule that helps in mediating leukocyte-trans-endothelial migration. VCAM-1 expression is absent in normal liver, but is strongly enhanced under inflammatory conditions (Volpes et al., 1992). LSECs constitutively express ICAM-1 along with hepatocytes, KCs and HSCs (Gulubova, 2005; Yin et al., 2007). The expression of ICAM-1 in LSECs is found to be upregulated by several inflammatory cytokines, such as TNF-α, IL-1β, or IFN-γ (Gangopadhyay et al., 1998; Oudar et al., 1998). A study was conducted on various endothelial cells isolated from the liver after transplantation rejection (Steinhoff et al., 1993). In LSECs, weak expression of ICAM-1 and ICAM-2 was observed under normal conditions. However, an elevated expression of ICAM-1 and ICAM-2 in LSECs were observed in chronic rejection as well as sepsis or viral infection cases. VCAM-1 was also shown to be partially upregulated in LSECs of irreversible and chronic rejection conditions. This study reflected the clinical relevance of sinusoidal endothelial adhesion molecules during liver transplantation.

LFA-1 (Lymphocyte Function Associated-1) belongs to the integrin family and is present on lymphocytes and leukocytes. It is an important component in the extravasation process mediating leukocyte and lymphocyte entry into the tissues from the bloodstream (Mitroulis et al., 2015; Walling and Kim, 2018). Binding of ICAM-1 expressed on LSECs to its ligand, LFA-1, expressed in pro-inflammatory cells mediates the migration of cells across the sinusoidal lining (Wong et al., 1997). This was one of the first studies to determine that Selectins, expressed on continuous vascular endothelium, are not an essential component for leukocyte transmigration into inflamed tissue of the liver. Similarly, a co-culture study conducted with LSECs and C26 tumor cells showed that LSECs expressing ICAM-1 mediate tumor migration (Benedicto et al., 2019). They also found an increase in the inflammatory IL-1β, IL-6, TNF-α, and PGE2 in the co-culture as compare to LSECs cultured alone. Interrupting the interaction between ICAM-1 and LFA-1 expressed on LSECs and C26, respectively, reduced the secretion of observed inflammatory molecules. This suggests the role of ICAM-1 and LFA-1 interaction in providing an inflammatory microenvironment suitable for colonization of tumor cells in the liver.

Another Ig superfamily adhesion molecule is PECAM-1 (CD31) (Newman et al., 1990). It is an adhesion molecule located at the cellular side and is involved in mediating endothelial cell-cell adhesion. CD31 is also responsible for carrying out leukocyte and monocyte trans-endothelial migration (Albelda et al., 1991). Whether LSECs express CD31 or not has long been debated and remains cryptic (Couvelard et al., 1993; DeLeve et al., 2004; Elvevold et al., 2008b). An upregulation in CD31 expression has been shown during the capillarization of LSECs in cirrhotic human liver (Couvelard et al., 1993). Also, its enhanced expression is detected in LSECs during focal nodular hyperplasia (Scoazec et al., 1995). Likewise, enhanced expression of CD31 has been related to the non-fenestrated and de-differentiated state of LSECs (DeLeve et al., 2006). Neubauer et al. (2000b) for the first time showed the expression of CD31 on LSECs and found no observable difference in CD31 expression under normal conditions and carbon tetrachloride-induced (CCl_4_) liver fibrosis. However, another study by the same authors demonstrated a constitutive expression of CD31 on LSECs, whereas, a decrease of CD31 was observed after CCl_4_ administration mediated by TNF-α *in vitro*. They hypothesized that reduced CD31 expression might aid in more desirable mononuclear cell transmigration (Neubauer et al., 2000a). Clearly, the on-going debate for the expression of CD31 on LSECs needs to be resolved and it should never be used as a marker for rodent LSEC purification.

## Perspectives and Conclusion

The past 35 years have proven to be a treasure-trove of discovery of the unique sinusoidal endothelium in liver. Advancements in their purification from rats and mice have enabled researchers to assess their physiological and biological role in normal and diseased phenotypes. Starting with the efforts of Seglen (1976) in the early 1970s and culminating to the current date, the procedure of dissociating the liver with collagenase-based enzyme mixtures have been very similar. The final steps in purification have involved differential centrifugation, fluorescent-activated cell sorting, and magnetic-activated cell sorting or a combination of these methods. A recent review on these techniques may be found in Meyer et al. (2016).

The anatomical shape and position of LSECs make them uniquely optimal for sequestering macromolecular materials from the blood. In this position, they act as “guardians” of the liver expressing numerous scavenger receptors and constantly monitoring the antigenic profile of the blood. As the liver is bathed in portal vein blood which drains the GI tract, there are many food and bacterial antigens flowing through in which LSECs play a major role in cleaning up and tempering other immune cells within the liver. Table 1 of this manuscript outlined many of the exogenous and endogenous ligands for all of these receptors. Redundancy in binding of multiple ligands shared by several receptors suggest the importance of physiological homeostasis with regards to external material coming into contact with blood and internal tissues. What is not known so much is the biochemistry of how these receptors are taking up multiple ligands at the same time or which amino acids/domains are interacting with each ligand. We think that this is an important step forward with the advancements in crystallography and cryo-EM methodologies. With an understanding of receptor-ligand interactions, pharmacological agents may be made to enhance or block these interactions according to required circumstances in the patient.

## Author Contributions

EP wrote the manuscript. AN wrote part of the manuscript and created the figures. EH edited and organized the manuscript with some brief writing in various sections. All authors contributed to the article and approved the submitted version.

## Conflict of Interest

The authors declare that the research was conducted in the absence of any commercial or financial relationships that could be construed as a potential conflict of interest.
